# Tick-borne zoonotic pathogens in birds in Guangxi, Southwest China

**DOI:** 10.1186/s13071-015-1249-8

**Published:** 2015-12-15

**Authors:** Jifei Yang, Zhijie Liu, Qingli Niu, Zhancheng Tian, Junlong Liu, Guiquan Guan, Guangyuan Liu, Jianxun Luo, Xiaolong Wang, Hong Yin

**Affiliations:** State Key Laboratory of Veterinary Etiological Biology, Key Laboratory of Veterinary Parasitology of Gansu Province, Lanzhou Veterinary Research Institute, Chinese Academy of Agricultural Science, Xujiaping 1, Lanzhou, Gansu 730046 P. R. China; Jiangsu Co-innovation Center for Prevention and Control of Important Animal Infectious Diseases and Zoonoses, Yangzhou, 225009 P. R. China; Center of Conservation Medicine & Ecological Safety, Northeast Forestry University, Harbin, Heilongjiang 150040 P. R. China

**Keywords:** *Borrelia garinii*, *Anaplasma phagocytophilum*, *Ehrlichia chaffeensis*, Bird, Tick-borne disease, Zoonosis, China

## Abstract

**Background:**

Wildlife is an important natural reservoir of many tick-borne pathogens. These agents have an impact on the health of humans and other animals throughout the world. This study was conducted to determine whether and what species of tick-borne agents had infected wild birds collected from Guangxi, in southwest China.

**Findings:**

Liver samples obtained from wild birds were tested for the presence of tick-borne pathogens by PCR assays and sequencing of the *flagellin* and 16S rRNA genes. *Borrelia garinii* was detected in Eurasian collared doves (2/57, 3.5 %) from among the 95 wild birds. *Anaplasma phagocytophilum* was detected in Eurasian collared doves (2/57, 3.5 %) and Eurasian eagle owls (2/13, 15.4 %). *Ehrlichia chaffeensis* and a potential novel *Anaplasma* sp. were identified in Common pheasant (1/12, 8.3 %). These results suggest the involvement of birds in the cycle of tick-borne diseases. To our knowledge, this is the first study to document infection of birds with *B. garinii*, *A. phagocytophilum*, *E. chaffeensis* and the novel *Anaplasma* sp. in China.

**Conclusions:**

Tick-borne zoonotic bacteria *B. garinii*, *A. phagocytophilum* and *E. chaffeensis*, and a potential novel *Anaplasma* sp., were identified in wild birds in southwest China. The presence of these agents in birds increases the potential spread over long distances and the risk of transmission of infection from birds to new hosts, including humans.

## Background

The transmission of infectious diseases between wild animals and humans is an issue of public interest [1]. It was reported that 71.8 % of human emerging infectious diseases (EIDs) identified between 1940 and 2004 originated from wildlife, and vector-borne diseases are believed to have been responsible for almost 30 % of EID events in the last few years [2].

Ticks can serve as vectors for a variety of pathogens, which are maintained in nature through an enzootic cycle between vector ticks and vertebrate hosts, primarily wild animals [3, 4]. Some of these agents, such as *Anaplasma phagocytophilum* and *Ehrlichia chaffeensis*, are now recognized as important emerging or re-emerging pathogens with serious public health implications [5, 6]. Growing evidence suggests that wild birds can alter the distribution of vectors and pathogens through their movement, and increase the infectious disease risk to wild and domestic animals as well as human beings [3, 7]. Therefore, it is important to identify wild birds that serve as major reservoirs of those agents. The purpose of this study was to determine the potential occurrence of *Borrelia burgdorferi* sensu lato, *A. phagocytophilum*, and *E. chaffeensis* in wild birds collected from Guangxi province, in southwest China.

## Methods

Wild birds that had died as a result of accidental injury were cryopreserved at the Guilin Wildlife Rescue Center in Guangxi Province, southwest China. Ninety-five birds were collected from March 2010 to April 2012 and identified as belonging to nine species: fifty-seven Eurasian collared doves (*Streptopelia decaocto*), eight Spotbills (*Anas poecilorhyncha*), twelve Common pheasants (*Phasianus colchicus*), thirteen Eurasian eagle owls (*Bubo bubo*), one Golden pheasant (*Chrysolophus pictus*), one Japanese wood pigeon (*Columba janthina*), one White-billed crow (*Corvus woodford*), one Chukar partridge (*Alectoris chukar*), and one Grey heron (*Ardea cinerea*). Liver samples were collected and preserved at −20 °C until use.

Total DNA was extracted from the 95 frozen liver samples using a Gentra Puregene DNA purification kit (Qiagen, Beijing, China) according to the protocols provided. All DNA samples were examined for the presence of *B. burgdorferi* s.l., *A. phagocytophilum* and *E. chaffeensis* by nested PCRs [4, 8–10]. Genomic DNA extracted from experimental sheep infected with *A. phagocytophilum* (Gene accession No. JN558811) and *B. garinii* strain PBi (American Type Culture Collection), and a plasmid containing the 16S rRNA gene of *E. chaffeensis* were used as positive controls; sterile water was used as the blank control. The amplification products were purified, cloned and subjected to sequencing (Sangon, Shanghai, China), and the nucleotide sequences obtained were compared with the published sequences in GenBank by a BLAST search.

The animal experiments complied with the Ethical Guidelines and were approved by our Institutional Ethics Committee.

## Results and discussion

The GenBank accession numbers for the partial 16S rRNA gene sequences obtained in this study were: KC916730 and KC916731 for *A. phagocytophilum*; KT596734–KT596736 for *E. chaffeensis*; and KT596739 and KT596740 for the unclassified *Anaplasma* sp. The partial flagellin gene sequences of *B. burgdorferi* s.l. were assigned accession numbers KT596741 and KT596742.

Of the 95 wild birds, two tested positive for *B. burgdorferi* s.l. (Eurasian collared dove, 2/57, 3.5 %). The flagellin gene sequences (377 bp) of *B. burgdorferi* s.l. obtained from Eurasian collared doves (GenBank accession no. KT596741 and KT596742) were 99.7 % identical. Sequence analysis revealed that the strains were most closely related to the *Borrelia garinii* genospecies (GenBank accession no. AB001716), with 99.7 to 100 % identity.

Infection with *A. phagocytophilum* was detected in two Eurasian collared doves (2/57, 3.5 %) and two Eurasian eagle owls (2/13, 15.4 %). The 16S rRNA gene sequences (641 bp) of *A. phagocytophilum* amplified from Eurasian collared doves (GenBank accession no. KC916730) and Eurasian eagle owls (GenBank accession no. KC916731) were 100 % identical to the sequences detected in deer and cattle from Japan (GenBank accession no. LC060987 and EU368728).

One liver sample collected from a Common pheasant (1/12, 8.3 %) was positive for *E. chaffeensis*. The partial 16S rRNA gene sequences (477 bp) of *E. chaffeensis* (GenBank accession no. KT596734–KT596736) were 99.0 % identical to those of strain Arkansas (GenBank accession no. NR_074500) and strains from ticks found in Southern China (GenBank accession no. AF147752). In addition, two sequences obtained from Common pheasant (GenBank accession no. KT596739 and KT596740) showed 99.8 % identity to the unclassified *Anaplasma* strains HLAE344 (GenBank accession no. GU075704) and BJ01 (GenBank accession no. JN715833) derived from *Haemaphysalis longicornis* ticks, suggesting a potential novel *Anaplasma* sp. in the Common pheasant.

These data revealed that four species of wild bird were positive for three tick-borne zoonotic pathogens: *B. garinii* was identified in Eurasian collared doves; *A. phagocytophilum* was identified in Eurasian collared doves and Eurasian eagle owls; *E. chaffeensis* and the potential novel *Anaplasma* sp. were identified in Common pheasants. No other bird species was positive for the pathogens investigated in this study.

To date, five genospecies (*B. burgdorferi sensu stricto*, *B. garinii*, *B. afzelii*, *B. sinica* and *B. valaisiana*) of *B. burgdorferi* s.l. have been identified in China [11, 12]. Among them, *B. garinii* is the genospecies most frequently isolated and is distributed mainly in northern China [12]. In the present study, we report for the first time the presence of *B. garinii* in birds in southwest China. Birds have been implicated as reservoirs for *B. garinii* and *B. valaisiana* through detection of these pathogens in ticks feeding on birds [7, 13–15]. Therefore, the occurrence of *B. garinii* in Eurasian collared doves is not surprising.

Seven species of *Ehrlichia* and *Anaplasma* (*E. chaffeensis*, *E. canis*, *A. phagocytophilum*, *A. ovis*, *A. marginale*, *A. centrale*, and *A. platys*) have been reported in China [16–19]. Among them, *A. phagocytophilum* and *E. chaffeensis* have been identified in ticks, rodents, deer, domestic animals and humans [19–22]. In this study, *A. phagocytophilum* and *E. chaffeensis* were identified for the first time in birds. Previous studies have shown low rates of infection with *A. phagocytophilum* and *E. chaffeensis* in ticks feeding on birds [7, 23]. This could be the reason for the low prevalence of these agents in birds in this study. Moreover, a potential novel *Anaplasma* sp. was identified in birds, which was distinct from all known *Anaplasma* species.

In summary, tick-borne zoonotic bacteria *B. garinii*, *A. phagocytophilum* and *E. chaffeensis*, and a potential novel *Anaplasma* sp., were identified in birds in this study. These results suggest the potential involvement of birds in the cycle of tick-borne diseases in southwest China. However, the role of these bird species as reservoir hosts for the agents identified should be further investigated.

## Conclusions

To our knowledge, this is the first study to document infection of birds with *B. garinii*, *A. phagocytophilum*, *E. chaffeensis* and the novel *Anaplasma* sp. in China. The presence of these agents indicates the possible role of birds in the dispersal of ticks and their associated infectious agents over large distances, and suitable vectors may allow the transmission of infection from birds to new hosts, including humans.
